# A New Synthetic Conduit for the Treatment of Peripheral Nerve Injuries

**DOI:** 10.1007/s00268-020-05620-0

**Published:** 2020-06-08

**Authors:** Selman Uranues, Georg Bretthauer, Gordana Tomasch, Dietmar Rafolt, Doris Nagele-Moser, Andrea Berghold, Reinhold Kleinert, Ivo Justich, Jörg Waldert, Horst Koch

**Affiliations:** 1 Section for Surgical Research, Department of Surgery, Medical University of Graz, Auenbruggerplatz 29, 8036 Graz, Austria; 2 Institute for Automation and Applied Informatics, Karlsruhe Institute of Technology, 76344 Eggenstein-Leopoldshafen, Germany; 3 Center for Medical Physics and Biomedical Engineering, Medical University of Vienna, 1090 Vienna, Austria; 4 Institute for Medical Informatics, Statistics and Documentation, Medical University of Graz, 8036 Graz, Austria; 5 Institute of Pathology, Medical University of Graz, 8036 Graz, Austria; 6 Clinical Division of Plastic, Aesthetic and Reconstructive Surgery, Medical University of Graz, 8036 Graz, Austria; 7 State Hospital for Neurology and Psychiatrics, 8055 Graz, Austria

## Abstract

**Background:**

Peripheral nerve defects (PND) often cause lifelong physical disability, and the available treatment options are often not satisfactory. PND are usually bridged with an autologous nerve transplant or a nerve guidance conduit (NGC), when coaptation as preferred technique is not possible. The aim of this experimental study was to determine the effectiveness of a novel NGC for regeneration in the treatment of PND.

**Materials and methods:**

A conduit made of gelatin with an innovative interior structure was tested for the repair of a 6-mm gap versus direct microsurgical suture repair without gap.

**Results:**

We found that bridging the defect with this conduit was as effective as direct microsurgical coaptation without a defect.

**Conclusions:**

This nerve conduit, effective in bridging neural defects, appears as an alternative to autologous nerve grafts, avoiding the problems related to nerve graft harvesting, host–donor differences in diameter, mismatches in number and pattern of fascicles, cross-sectional shape and area, and morbidity of the donor area.

## Introduction

Peripheral nerve defects are most often due to trauma and have a high potential for major disability [1]. Annually more than 200,000 nerve injuries are treated surgically in the USA [2]. Younger men are the most prone to these injuries (male/female ratio 3:1, mean age 32–35 years) [3–5]. Thus, people in the most productive phase of their lives are affected leading to high treatment and socio-economic costs [6].

Coaptation with direct suture repair is optimal for a good functional result but is only possible for defects of less than 5 mm in length, as larger gaps may cause tension on the suture line leading to poorer results [7, 8]. In this setting, autologous nerve grafts are being used serving as scaffolds supplying nutrients, while the basal lamina, endoneural tubes, and Schwann cells guide the regenerating axons to the distal stump [9, 10]. However, nerve grafting can result in sensory defects at the original supply areas with potentially subsequent neuromas and chronic pain. Furthermore, differing nerve diameters and lengths may not allow an optimal fit; thus, nerve guidance conduits (NGC) have been proposed to bridge the defect [11–13]. Their use with large nerves is limited since they have been reported to fail to support regeneration [13, 14]. In a pilot study [15], we described a new nerve conduit to circumvent the known deficiencies of NGCs and improve nerve regeneration.

The aim of the present study was to determine the effectiveness of neural regeneration comparing prosthetic repair of a 6-mm nerve gap versus direct microsurgical suture repair of the same type of nerve injury but without a gap in porcine sciatic nerve. The test hypothesis was that the clinical result with conduit repair for a nerve injury with a gap would be as good as direct microsurgical coaptation without a gap.

## Materials and methods

After skin incision, mini-pigs were randomized to one of three groups with a ratio of 2:2:1 (Table 1). The left sciatic nerve was completely severed in the paravertebral segment before its first branch. After transection, the nerve was repaired with either standard microsurgical coaptation (group I) or implantation of the experimental conduit leaving a 6-mm gap between the proximal and distal stumps (group II). Group III served as controls: the nerve was severed and left as it was.

**Table 1:** Study groups after randomization

	*n*	%
Suture repair	13	37
Prosthetic repair	15	43
Controls	7	20
Total	35	100

During the 10-month study period, the animals kept in small groups received optimized special food to minimize weight gain and body growth.

Before the nerve transection and postoperatively at 8-week intervals, all animals underwent electrophysiological studies and muscle ultrasound and were induced to walk a defined, uniform stretch for video documentation (Table 2).

**Table 2:** Baseline characteristics of the study

Examination (visits 1–6)	Time	Procedures
V1	Preoperative	VID, US, EMG, PD, randomization, followed by surgery I
V2	8 weeks postop.	VID, US, EMG, WC
V3	16 weeks postop.	VID, US, EMG, WC
V4	24 weeks postop.	VID, US, EMG, WC
V5	32 weeks postop.	VID, US, EMG, WC
V6	40 weeks postop.	VID, US, EMG, WC, PD, followed by surgery II

Electromyography (EMG), photo documentation (PD), ultrasound of the target muscle (US), video for gait analysis (VID), wound check (WC)

At the end of the 10-month observation period, the final physiological tests and videos were taken. The animals were sacrificed according to study guidelines, and the nerve anastomosis was resected at proximal and distal distances of 2 cm for morphologic and histological study.

**Fig. 1:**
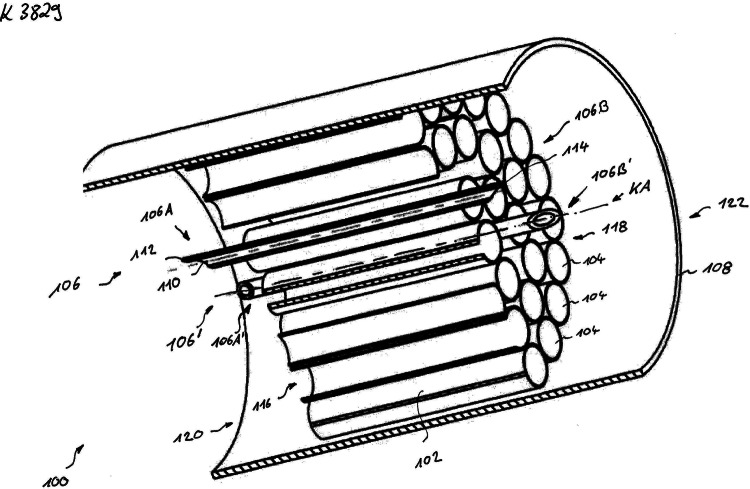
Scheme of the conduit

The conduit is composed of biocompatible absorbable gelatin [15]. The basic body is made of biocompatible absorbable gelatin with a length of 18 mm. The middle third of the prosthesis contains a bundle of gelatin microtubules fixed with a similar gelatin adhesive and three evenly distributed, hollow, miniature titanium rods. The titanium rods extend 1 mm into both ends in order to enter the severed nerve stumps providing stability within the tube (Fig. 1).

### Perioperative treatment

Food but not water was withheld for 12 h before surgery. General anesthesia and postoperative analgesia were performed according to standard protocol (Appendix). Antibiotic treatment (amoxicillin/clavulanic acid) was administered before skin incision and continued orally for 5 days during the recovery period.

### Surgical technique

**Fig. 2:**
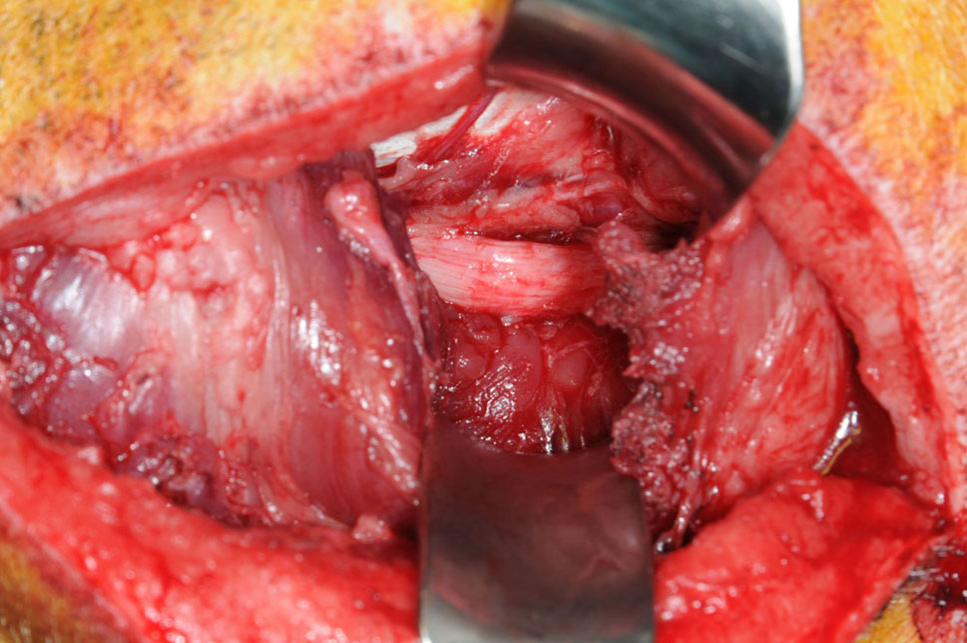
Exposure of the sciatic nerve before its first branches

**Fig. 3:**
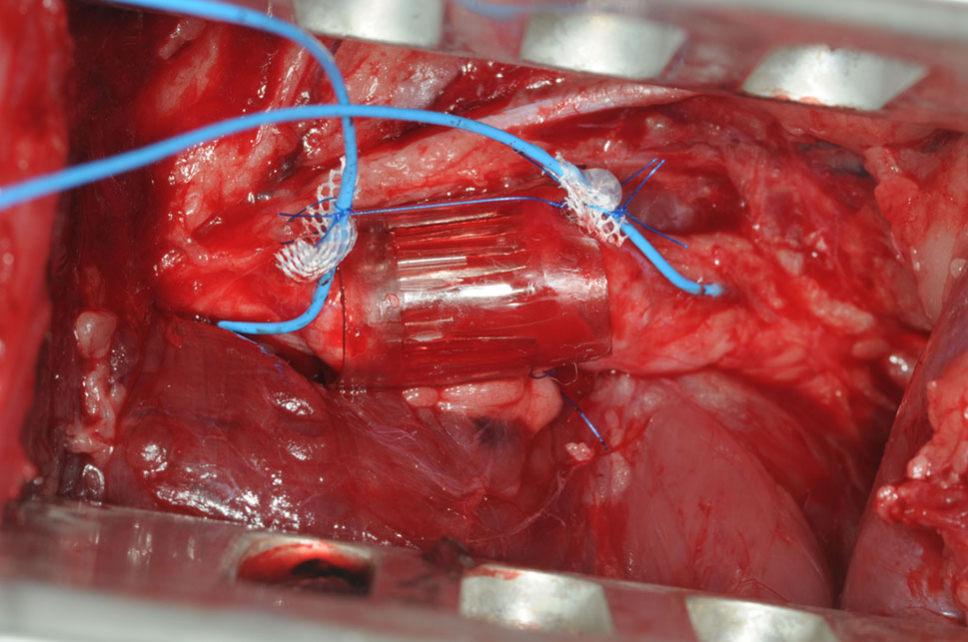
The sciatic nerve shown in Fig. 2 after conduit repair and positioning of two monopolar electrodes proximal and distal of the injury site

**Fig. 4:**
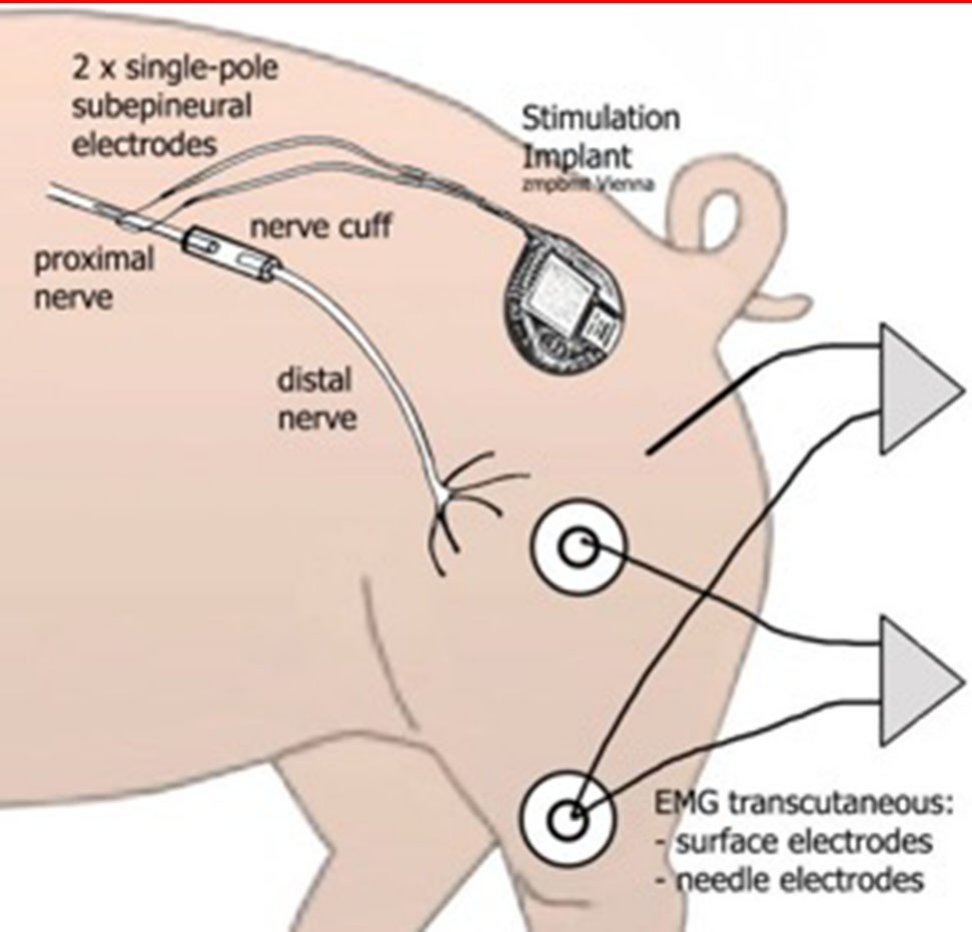
Experimental setup

After exposure of approx. 2.5 cm of the sciatic nerve (Fig. 2), two monopolar electrodes (Streamline ^®^, Medtronic, Minneapolis, MN) were positioned proximal to the planned nerve division on the anterior and posterior surface of the nerve to reach a maximum number of nerve fibers (Fig. 3). Then, they were moved in opposite directions for 4 mm along the nerve axis to produce an electric field vector length to the nerve and then inserted under the epineurium and fixed with non-absorbable 9-0 sutures. A stimulation device (max. 10 mA, programmable by transcutaneous magnetic telemetry [16]) was implanted subcutaneously in a protected area on the back of the animal (Fig. 4).

EMG electrodes were applied to the semitendinosus muscle, the largest target muscle supplied by the sciatic nerve. After baseline electrophysiological evaluation (V1), the nerve was sharply severed at the previously marked site. Complete transection was verified by repeat electrophysiological testing.

In the group I, microsurgical coaptation was performed by precisely adapting the fascicle groups with Ethilon (Ethicon, Somerville, NJ) 9-0 epi-/perineural interrupted sutures.

In group II, the nerve ends were inserted proximally and distally into the conduit. The titanium rods extended 1 mm into both ends of the severed nerve. The gap of 6 mm between the stumps was bridged with the middle section of the prosthesis filled with microtubules. The two ends of the prosthesis were each fixed to the nerve with an epineural 4-0 prolene suture (Ethicon, Somerville, NJ).

### Electrophysiological tests

Electrically evoked potentials (EEPs) of the target muscle were taken to evaluate nerve recovery. Needle electrodes were inserted transcutaneously for the EMG. For a more integrated EMG response, additional surface electrodes were attached on the skin over the muscle belly. The recorded m-waves (KeyPoint^®^ 2-channel electromyograph; Alpine Biomed Inc.) represented the synchronized EMG response of all muscle fibers supplied by the specific stimulated nerve bundle via the subepineural implanted electrodes described above. In this electrode setup, the electrical field addresses a maximum number of nerve fascicles with a minimum of stimulation amplitude.

During the test, the amplitude was increased by increments of 0.2 mA up to a maximum of 5 mA in order to assess the twitch threshold and saturation voltage of the recorded m-wave signal. The threshold of the muscle contraction was determined by palpation and visual inspection (Table 3).

**Table 3:** Initial m-wave from surface EMG and needle EMG

	*A*		*P*		*C*	
EMG	Mean	STD	Mean	STD	Mean	STD
Surface	28.3	8.7	26.6	8.1	29.0	3.0
Needle	28.2	7.1	22.9	5.9	22.9	5.3

m-wave response (mV): *A* coaptation, *P* prosthesis, *C* control

### Analysis of muscle atrophy

The diameter of the target muscle was measured sonographically at baseline and at 8-week intervals for the duration of the study. To assure standardized measurements, the measurement site was marked with a tattoo. The decrease in muscle mass due to atrophy and subsequent increase with recovery were documented and given a score. The severity of muscle atrophy was recorded on a numeric rating score ranging from 0 to 18. This atrophy score was created by the authors in order to stratify the gradation depending on the thickness and echo intensity of the muscle. Scores of 9 or less were counted as “poor,” 10–14 as “moderate,” and 15–18 as “good.”

### Gait analysis

All gait videos were recorded with the same camera (Sony HDR-UX19) during the previously described induced walking stretch. After 10 months, the videos were rated by two independent researchers, otherwise uninvolved in the study using the 10-grade Miami Porcine Walking Scale (MPWS) [17, 18].

### Morphological and histological assessment

The morphological studies focused on three areas: the proximal nerve stump, the midsection including the area of the prosthetic bridge in the respective group, and the distal nerve stump. The outcome was based on the degree of neuroma formation and remyelinization, as well as reactive inflammatory changes. Since there is no other gradation for degenerative changes of pig nerves, this gradation was adapted by the neuropathologist according the neuropathological scoring of degenerative changes in humans. The individual animals were scored with grades I–III. The histology techniques used were hematoxylin and eosin stain, Masson’s trichrome stain and immune histochemistry.

### Statistical methods

The documented changes in parameters were evaluated with the analysis of covariance: group differences by analysis of variance, Kruskal–Wallis test, or Chi-square test as appropriate. Morphological results were assessed with Fisher’s exact test and presented in a contingency table.

This study was approved by the Austrian Federal Ministry of Science, Research and Economy (Authorization Nr. BMWF-66.010/0082-II/3b/2010) as well as by the Institutional Review Board of the Medical University of Graz. All animal procedures were performed in accordance with Austrian and European law (Directive 2010/63/EU) which are covering all requirements listed in the ARRIVE Guidelines (Animal Research: Reporting In Vivo Experiments).

## Results

Thirty-five healthy female Göttingen mini-pigs (average age 14 months, average weight 27.7 kg [SD 4.7]) were included. All animals survived the 10-month study period and underwent the electrophysiological function tests and gait analyses. Following randomization during surgery, 13 animals were in the coaptation group (group I), 15 in the conduit group (group II) and seven in the control group (group III).

### Electrophysiological assessment

**Fig. 5:**
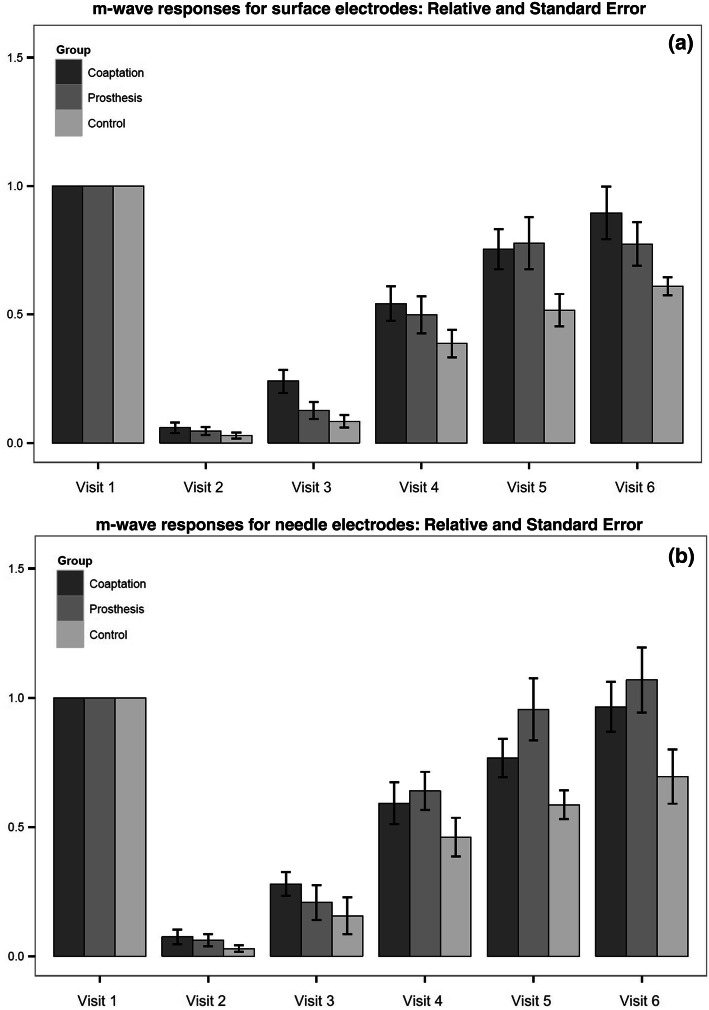
**a**, **b** Process of recovery in relation to the initial values for all groups and both electrode types. Visit 1 shows m-wave measurements before nerve transection. Visits 2–6 are follow-up measurements. All values are related to the first visit in each group

The most important parameter reflecting nerve recovery was the m-wave amplitude with supra-maximal stimulation input (Fig. 5a, b). The mean initial values (Visit 1—V1) of the maximal m-wave amplitude before transection of the nerve are shown in Table 1 for the surface electrode and needle electrode, respectively. Group I showed a tendency of a faster recovery process at the beginning (V2–4), but later (V5, 6) there was no statistically significant difference (*p* value V5: 0.865; V6: 0.372) found between groups I and II (80% of the initial values). The group II showed a delay at the beginning of the recovery process, possibly due to the 6-mm gap. The control group showed smaller EMG values throughout follow-up and ended at a level of 60%.

In general, there were differences in the growth speed of the fibers resulting in a higher standard deviation/mean quotient in the early growth period. This parameter was much higher for group II at V2 but ended at the same value as the coaptation group (I).

### Ultrasound muscle volume assessment (Fig. 6)

At 8 weeks (V2) the muscle was maximally atrophied in all groups; thereafter, continuously volume recovery began. At the end of the study period, muscle volume in all three groups was similar to baseline. Of note, the values in the nerve prosthesis group were more coherent (i.e., grouped more closely together) than in the coaptation group, where they were clearly divergent. In general, the muscle volume behaved similarly to the body weight of the animals. Animals with reduced muscle volume at the end of the observation period had also scarcely gained weight. There was no significant difference in muscle volume recovery (*p* = 0.59) between groups.

**Fig. 6:**
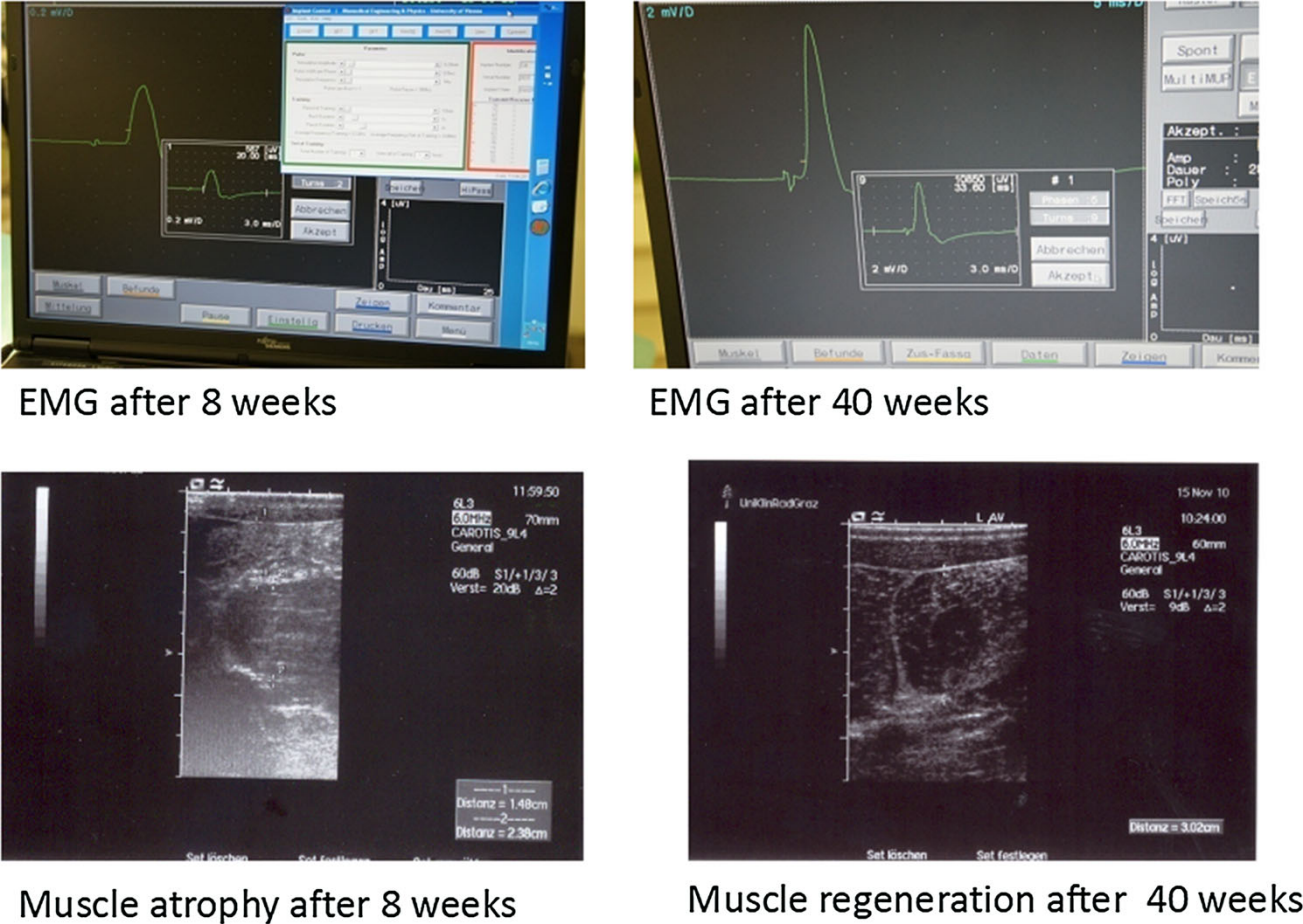
EMG and ultrasound measurement of the muscle

### Gait analysis

Gait analysis (Tables 4, 5) clearly showed impairment after 8 and 16 weeks; notwithstanding, the prosthesis group tended to have better initial gait patterns than the other groups. At the end of the study, the animals in all three groups achieved a score of more than 90% of the baseline value and thus could walk almost normally. Animals with the conduit appeared to have slightly better gait patterns than those with coaptation.

**Table 4:** Miami Porcine Walking Scale (MPWS) [Kuluz, S 3]

Grade	Description
1	No movement
2	Movement of hips only (hind limbs move in phase)
3	Movement of hips and knees
4	Rhythmic flexion/extension movement of all joints but without weight support
5	Attempts weight bearing but cannot support weight on hind limbs, drags hind limbs
6	Occasional weight bearing on hind limbs, drags hind limbs
7	Stands, attempts to walk, no alternating hind limb movement
8	Stands, walks 3–5 steps, some alternation of hind limbs but poor fore-hind limb alternation
9	Walks 5+ steps with alternation of hind limbs and with fore-hind limb alternation, limited knee flexion, some dragging of hooves
10	Walks 5+ steps with alternation of hind limbs and with fore-hind limb alternation, good knee flexion (normal walking)

**Table 5:**
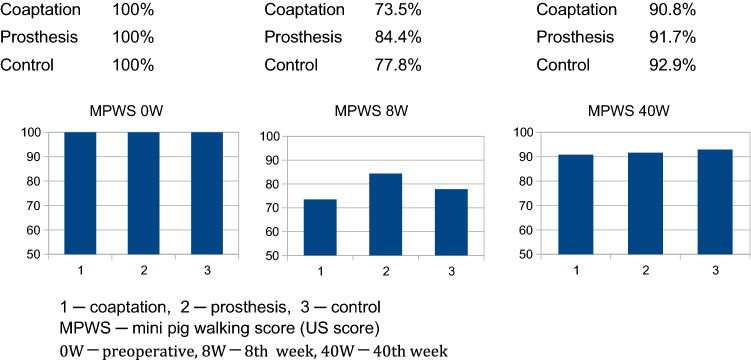
Results of the mini-pig walking score (MPWS)

### Morphological and histological assessment

The individual animals were scored with grades I to III (Tables 6, 7). In all cases, a slight tendency to neuroma formation was seen as a positive factor and was scored as grade III; the same was true for the tendency in percentage toward a higher degree of central remyelinization. On the proximal stump, the main feature was the tendency toward endoneural fibrosis as is seen with neuritis, whereas on the distal stump, it was mainly the reduction in nerve fibers, the thickness of the myelin sheath, and signs of fibrosis. A low tendency to granuloma formation was seen to indicate a positive effect on the outcome and was graded III, whereby no or only slight inflammatory reaction in the form of suture abscess formation or endoneuritis was also graded III.

**Table 6:** Characteristics of the morphological investigations

	I (1)	II (2)	III (3)
Degree of neuroma formation	High	Moderate	Slight
Central remyelinization	Slight, 5–20%	Moderate, up to 30%	High, up to 50%
Granuloma formation	High, with abscess formation	Moderate	Slight
Proximal stump: endoneural fibrosis	High, with neuritis	Moderate	None
Distal stump: reduction of nerve fibers and thickness of myelin sheath with fibrosis and inflammation	High	Moderate	Slight
Peripheral muscle: neurogenic atrophy	Pronounced (distinct panfascicular formation of atrophic fiber fields; lipomatosis)	Moderate (perifasicular fields)	Discrete (angular fibers)
Point score	6–9	10–14	15–18
Score interpretation	Poor	Moderate	Good

**Table 7:** Morphological test results

Groups	Coaptation	Conduit	Control	Total	Fisher’s exact test
*Score*					
6–9: poor	3	3	3	9	0.800
10–14: moderate	7	10	4	21	
15–18: good	3	2	0	5	
Total	13	15	7	35	

Histological study of the muscles showed no significant difference with respect to neurogenic atrophy, whereby the atrophy grades 1–3 (1—slight, 2—moderate, and 3—severe) were evenly distributed in the coaptation group, while the atrophy grades in the nerve prosthesis group were mainly in the moderate range.

Concerning muscle atrophy, most of the animals were scored as “moderate,” with the conduit group in the lead with 66.6%. Only five (14.7%) of the animals rated “good” (Tables 6, 7).

Overall, all results in the animals with nerve-gap injuries treated with the conduit were as good as those animals whose severed nerve ends were coapted end to end without a gap. Interestingly, the results in the control group, whose nerve ends were left in their natural position after the nerve was severed, were remarkably good.

## Discussion

Nerve regeneration is primarily driven by the ability of the surviving neurons to form axonal sprouts that grow from the proximal stump to the distal stump to reinnervate their original targets [10]. Tension-free coaptation of the severed nerve ends with as little time loss as possible is the current treatment of choice [19]. Most traumatic nerve injuries, however, present with a defect of more than 5 mm requiring bridging. Autologous nerve grafting has serious limitations as little nerve tissue is available in the human body and the retrieval procedure is prone to complications. The harvested donor nerve tissue leaves a functional deficit at the explant site and must also match the recipient nerve in size, diameter, and structure. For these reasons, preference is increasingly given to biological or synthetic conduits. Currently, biodegradable materials such as chitosan, collagen, polyglycolic acid, and polyglactic acid are mainly used [8, 13]. As yet, there has been no breakthrough and conventional direct suture repair and autologous nerve grafting are still the gold standards [8, 13, 20–22]. These techniques, however, require specialists and thus often transferable to specialized centers, which might delay treatment.

Time is a critical factor to promote axonal regeneration and to minimize the period of Wallerian degeneration [9]. Time lost thereby can adversely affect functional recovery [13, 19]. Our aim was to develop a conduit that can be easily implanted without special equipment by any general or trauma surgeon. The technique is simple, straightforward, and not time consuming.

In the choice of materials for nerve guidance conduits, resorbability and biocompatibility, especially as reflected in non-provocation of an inflammatory response, are of utmost importance and the gelatin we chose is optimal in this respect. No animal, either in the preliminary or the main study, showed any intolerance [15]. Our study leads to believe that the outer sheath of the conduit and the microtubules, both made of gelatin, provide a route for the budding and elongating axons to follow that mimics the natural structure of the neural pathway, with endoneural connective tissue covering the individual fascicles, and the individual nerve fibers being stabilized and protected from ingrowth of external connective tissue. The titanium rods extending 1 mm into the nerve stumps steady the prosthesis and the nerve ends to ensure a stable straight-on connection. There was no histological evidence that the titanium rods caused any additional damage. Apparently, little, if any, attention has been given so far to the stabilization of the nerve lesion under repair. Indeed, immobilization is highly recommended for injury repair in general and should promote better nerve regeneration.

The molecular and cellular processes involved in nerve regeneration are best revealed with morphological studies, which showed that nerve healing proceeded just as well when the defect was bridged with our conduit as with direct coaptation.

This study was designed to closely resemble the human situation and in that it comprised a neurophysiological study that monitored all phases of nerve regeneration over the entire 10-month period. Ultrasound studies proved to be an easy and practicable way to determine the volume of the target organ, the semitendinous muscle, and the degree of muscle recovery served to confirm the results of the neurophysiological tests.

Interestingly, the control group achieved remarkable good results in which the transected nerve ends were not treated. Even if there is no scientific explanation for it the capacity for self-healing in mice and rats is well described. It is our suggestion that the good preserved clean nerve ends close to each other was potentially the reason for this result.

There were several limitations in this study. The first limitation was the methodology used, since the outcome of two different types of injuries, with and without gap, was compared. Our goal was to determine the equivalence of the new conduit in a more challenging nerve injury with a gap, versus the standard coaptation treatment without gap. Although the physiology of nerve regeneration in these two different repair methods might be different, we were driven by the high clinical incidence of injuries with gap that need surgical repair.

The second limitation was the electrophysiological tests used in this study. Both, electrically evoked potentials (EEPs) and the recorded m-waves for the electromyogram are based on human methodologies. Since there is no published research on this methodology on mini-pigs, its correctness can be assumed but not confirmed.

The same limitation is also valid for the muscle atrophy analysis, and for the understanding of the morphological and histological changes at the surgery sites. As mentioned above, our study group created the physiological scores according to the well-known gradations of human tissues.

Lastly, the main limitation of the shown technique is that these promising results have been achieved in an animal experiment and cannot be automatically transposed to human conditions. Properly controlled studies with human subjects are essential to translate the experimental results into clinical application.

## Conclusion

We present a nerve conduit made of gelatin with a microtubular structure in the middle third of the prosthesis and stabilizing titanium micro-rods. This allows neural regeneration and functional results in the setting of nerve repair with a physical gap.

This neural prosthesis may provide an alternative to autologous nerve grafts, thus avoiding the problems and morbidity related to nerve graft harvesting.

The shown technique can be easily learned by general and trauma surgeons called upon to repair such nerve injuries and can help to reduce the time between the trauma and repair of the injured nerve.

## Appendix: Anesthesia and analgesic protocol

Premedication consisted of intramuscular (IM) azaperone 2 mg kg^−1^, midazolam 0.5 mg kg^−1^, ketamine 15 mg kg^−1^, and butorphanol 0.3 mg kg^−1^. Carprofen 4 mg kg^−1^ was given IM for preemptive analgesia. General anesthesia was induced with propofol 2–5 mg/kg intravenously (IV). After endotracheal intubation and connection to a rebreathing circle system, volume-controlled ventilation (10^−15^ ml kg^−1^) was started. Anesthesia was maintained with sevoflurane (1.5%) in 80:20 oxygen/air (1.5 l min^−1^ flow) and 20 µg kg^−1^ h^−1^ fentanyl constant rate infusion (CRI). Heart rate, oxygen saturation, end tidal carbon dioxide, and rectal temperature were monitored during the whole procedure and maintained within normal limits. Ringer's solution was infused with 10 ml kg^−1^ h^−1^ IV, and 0.6 mg kg^−1^ h^−1^ ketamine SSRI was given to reduce neuropathic pain. Immediately before nerve dissection, a 1.5 mg g^−1^ lidocaine bolus was given IV for additional analgesia. Postoperative analgesia consisted of methadone (0.1 mg kg^−1^, IM), tramadol (3 mg kg^−1^, IM), carprofen (4 mg kg^−1^, IM), and transdermal fentanyl (100 µg h^−1^).

To prevent or reduce neuropathic pain, Gabapentin 300 mg was given orally for 10 days.
